# Altered functional responses by PAR1 agonist in murine dextran sodium sulphate-treated colon

**DOI:** 10.1038/s41598-022-21285-2

**Published:** 2022-10-06

**Authors:** Tae Sik Sung, Suk Bae Moon, Brian A. Perrino, Kenton M. Sanders, Sang Don Koh

**Affiliations:** 1 Department of Physiology and Cell Biology, University of Nevada School of Medicine, Reno, NV 89557 USA; 2 Present Address: Biomedical Research Institute, Seoul National University Hospital, Seoul, 03080 South Korea; 3 Present Address: Department of Surgery, Kangwon National University School of Medicine, Chuncheon, 24341 South Korea

**Keywords:** Physiology, Gastroenterology

## Abstract

Protease-activated receptor-1 (PAR1) is highly expressed in murine colonic smooth muscles. Responses to PAR1 activation are complex and result from responses in multiple cell types. We investigated whether PAR1 responses are altered in inflamed colon induced by dextran sodium sulfate (DSS)-treatment. Colitis was induced in C57BL/6 mice by administration of 3% DSS in drinking water for 7 days. Measurements of isometric force, transmembrane potentials from impaled smooth muscle cells, quantitative PCR and Western blots were performed. Thrombin, an activator of PAR1, caused transient hyperpolarization and relaxation of untreated colons, but these responses decreased in DSS-treated colons. Apamin caused depolarization and increased contractions of muscles from untreated mice. This response was decreased in DSS-treated colons. Expression of *Kcnn3* and *Pdgfra* also decreased in DSS-treated muscles. A second phase of thrombin responses is depolarization and increased contractions in untreated muscles. However, thrombin did cause depolarization in DSS-treated colon, yet it increased colonic contractions. The latter effect was associated with enhanced expression of MYPT1 and CPI-17. The propagation velocity and frequency of colonic migrating motor complexes in DSS-treated colon was significantly higher compared to control colons. In summary, DSS treatment causes loss of transient relaxations due to downregulation of SK3 channels in PDGFRα^+^ cells and may increase contractile responses due to increased Ca^2+^ sensitization of smooth muscle cells via PAR1 activation.

## Introduction

The prevalence of inflammatory bowel disease (IBD) has increased in recent years, and leads to colonic dysmotility^1,2^. Colonic motor disturbances result from changes in neuromuscular components, including enteric neurons, smooth muscle cells (SMCs), interstitial cells of Cajal (ICC), platelet-derived growth factor receptor α positive (PDGFRα^+^) cells. The latter 3 cellular components form the SIP syncytium^3^ because the cells are electrically coupled and provide pacemaker activity that generates patterned contractions and integrate neural inputs from enteric motor neurons^4,5^. ICC are decreased in number, experience morphological changes and become defective in several motility disorders^6–8^. Although substantial progress has been made in understanding inflammatory colonic dysmotility, little is known about the effects of inflammation on the components of the SIP syncytium. This study focused on contributions of PDGFRα^+^ cells in response to PAR1 activation in inflamed colon.

PDGFRα^+^ cells (aka ‘fibroblast-like cells’) are distributed in the circular and longitudinal muscle layers and in the region between the muscle layers in proximity to the myenteric plexus^9^. These cells express small conductance Ca^2+^-activated K^+^ channels (SK3 channels encoded by *Kcnn3*)^9,10^. Activation of SK3 channels by dynamic changes in cytoplasmic Ca^2+^ can initiate spontaneous transient outward currents (STOCs) and hyperpolarization of PDGFRα^+^ cells and the electrically coupled cells in the SIP syncyitum^9,10^ SK channel antagonists, such as apamin, induce depolarization of GI muscles via inhibition of the outward currents mediated by SK channels^9–13^.

Previous studies have shown that Protease-activated Receptor (PAR) agonists alter the excitability of GI SMCs via complex interactions with cells of the SIP syncytium^14,15^. PARs activate SK3 channels in PDGFRα^+^ cells to elicit inhibitory effects, and excitatory responses are elicited by PAR agonists and mediated largely by ICC^14^. PAR2-induced relaxation of colonic smooth muscle was reported to be impaired after dextran sodium sulphate (DSS)-induced colitis^16^. We found that PAR1 (F2r) is highly expressed in PDGFRα^+^ cells in the colon^14^ and activation of PAR1 causes complex response consisting of initial relaxation followed by contraction^14^. We hypothesized that changes in SK3 channel expression and/or defects in PDGFRα^+^ cells may affect the excitability of SMCs in inflamed colonic smooth muscles. Thus, we examined whether *Pdgfra* or *Kcnn3* expression is downregulated in PDGFRα^+^ cells in DSS-treated colon, causing the excitatory effects of PAR1 agonist to become dominant.

## Results

### DSS-induced colitis model

**Figure 1:**
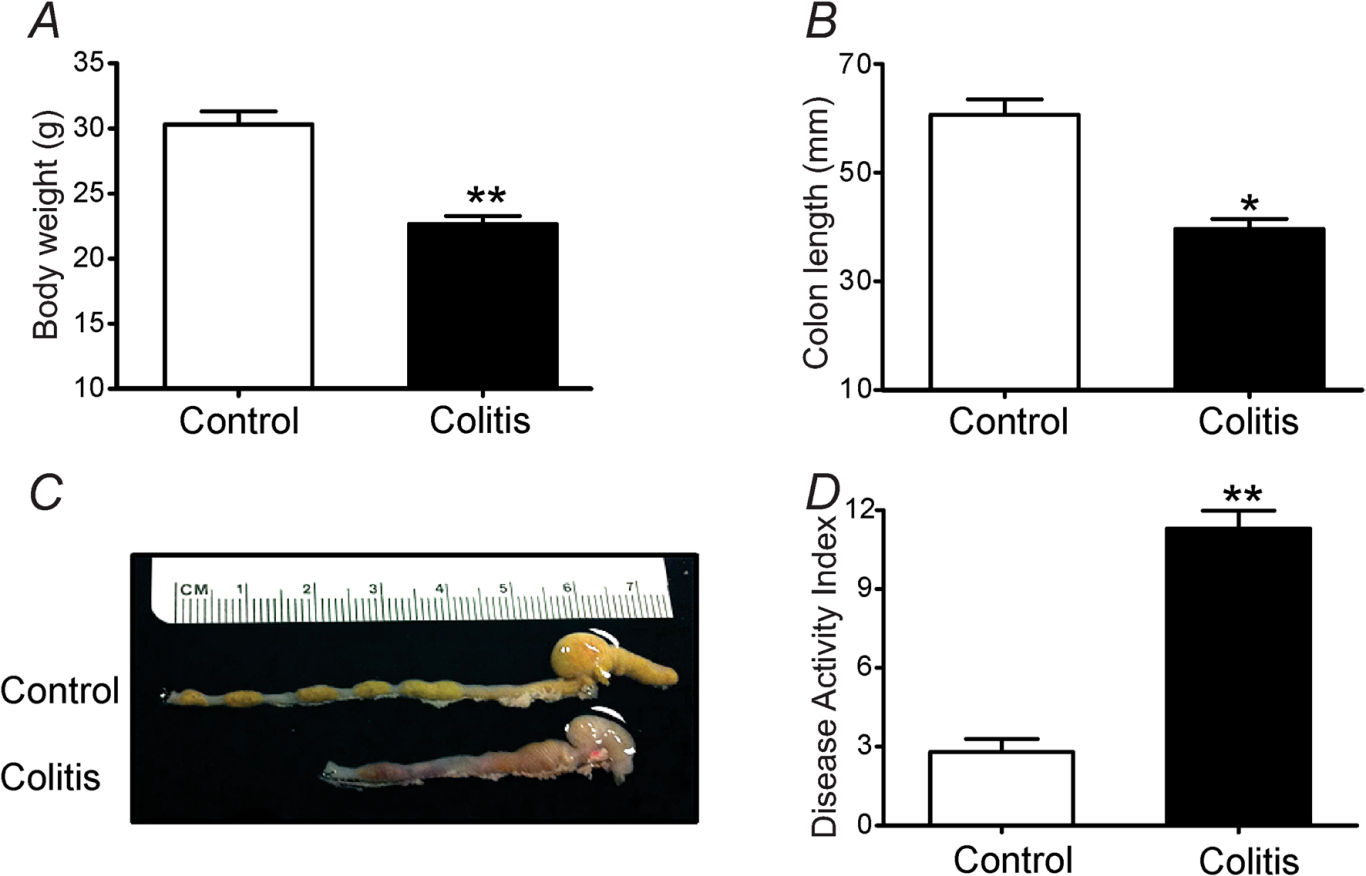
Comparison of body weight, colon length and Disease Activity Index in control and DSS-treated colons. (**A**) Body weight of colitis mice was significantly decreased at 7th day. (**B,C**) Averaged total length of DSS-treated colon was shorter compared to control colon. (**D**) DAI was increased in DSS colitis at 7th day. * and ** in panels (**A,B,D**) denote *P* < 0.05 and *P* < 0.01 by unpaired t-test.

After DSS treatment, body weight significantly decreased by day 7 (30.3 ± 1.0 g in control mice vs 22.7 ± 0.6 g in colitis mice; *P* < 0.01; n = 23; Fig. 1A). Average total length of colons also decreased after DSS-treatment (39.6 ± 1.9 mm, n = 11) compared to control (60.7 ± 2.8 mm, n = 9, *P* < 0.05, Fig. 1B,C). The disease activity index (DAI; see description in Methods section) was increased in DSS colitis (*P* < 0.01; n = 7; Fig. 1D).

### The effects of thrombin on resting membrane potential

**Figure 2:**
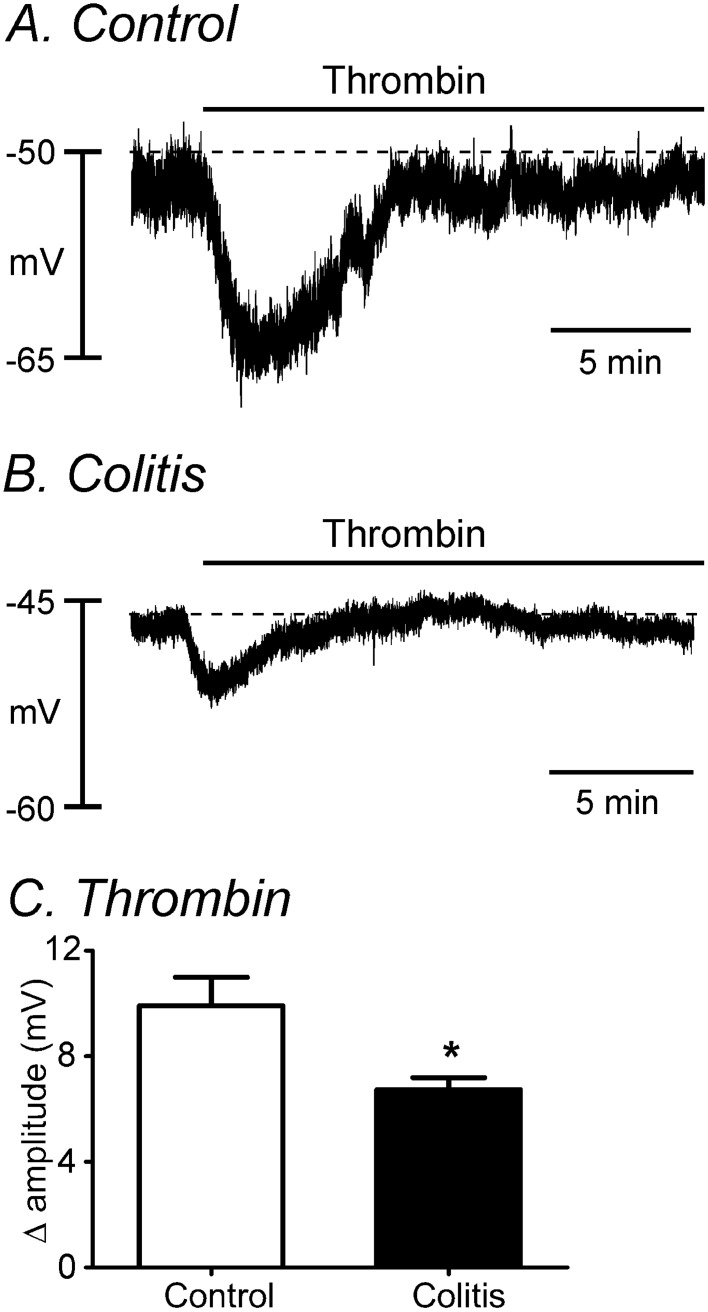
Comparison of thrombin responses on the membrane potentials in control and DSS-treated colons. (**A,B**) Thrombin hyperpolarized colonic muscles in control colon (**A**) and induced less hyperpolarization in DSS-treated colon (**B**). (**C**) The summarized data showed the changes in the amplitude of hyperpolarization by thrombin. *Denotes *P* < 0.05.

Colonic smooth muscle cells (SMCs) were depolarized after DSS treatments to − 45.7 ± 2.0 mV (n = 7), as compared to membrane potentials in SMCs of control (i.e. untreated) colons (− 53.1 ± 1.2 mV, n = 13, *P* < 0.05). Thrombin (50 U/ml) caused transient hyperpolarization of SMCs in colons of untreated and DSS-treated mice. Thrombin hyperpolarized SMCs from − 50 ± 1.4 mV to − 60.4 ± 2.3 mV (Fig. 2A) and from − 43.8 ± 2.1 mV to − 50.6 ± 2.3 mV in untreated and DSS-treated colons, respectively (Fig. 2B). The magnitude of hyperpolarization caused by thrombin was reduced in DSS-treated colons in comparison to untreated colons (Δ 9.9 ± 1.1 mV vs Δ 6.7 ± 0.4 mV, n = 5 each, *P* < 0.05, Fig. 2C). Thus, PAR1-mediated hyperpolarization was partially impaired in DSS-treated colons.

### The effects of thrombin on spontaneous contractions

**Figure 3:**
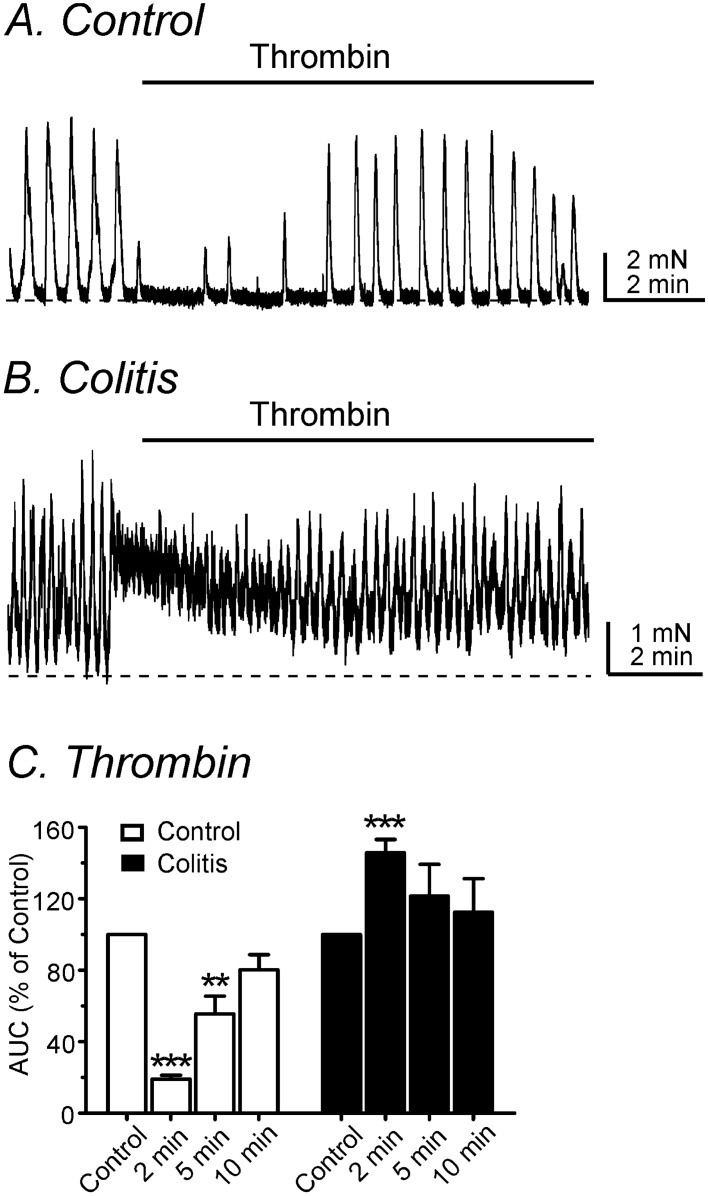
Mechanical responses by thrombin in control and DSS-treated colon. (**A**) Thrombin (50 U/ml) decreased spontaneous contractions initially followed by contractions gradually in control colons. (**B**) Thrombin induced initial contractions without relaxation in DSS-treated colons. (**C**) The summarized data of changes in the AUCs of contractions after thrombin application as a function of time (up to 10 min). ** and ***Denote *P* < 0.01 and *P* < 0.001, respectively.

The area under the curve (AUC) of spontaneous contractions were analyzed before and after treatment with thrombin. Thrombin (50 U/ml) transiently decreased spontaneous contractions of control colons, and contractions recovered gradually. In contrast, thrombin elicited only excitatory responses in DSS-treated colons. Responses to thrombin consisted of rapid phasic contractions superimposed upon tone and no initial relaxation phase was observed after DSS-treatment. AUCs describing contractile responses within 2 min of thrombin treatment were 19.1 ± 2.2% in control and 145.8 ± 7.3% in DSS-treated colon, respectively (*P* < 0.01, n = 6 and 7, respectively; see Fig. 3). AUC% was calculated by normalization of each control condition (before addition of thrombin). The results show that PAR1-mediated relaxation was impaired, and the excitatory phase of the PAR1 response became dominant in DSS-induced colitis.

### Effect of SK3 antagonist on colonic membrane potential and spontaneous contractions

**Figure 4:**
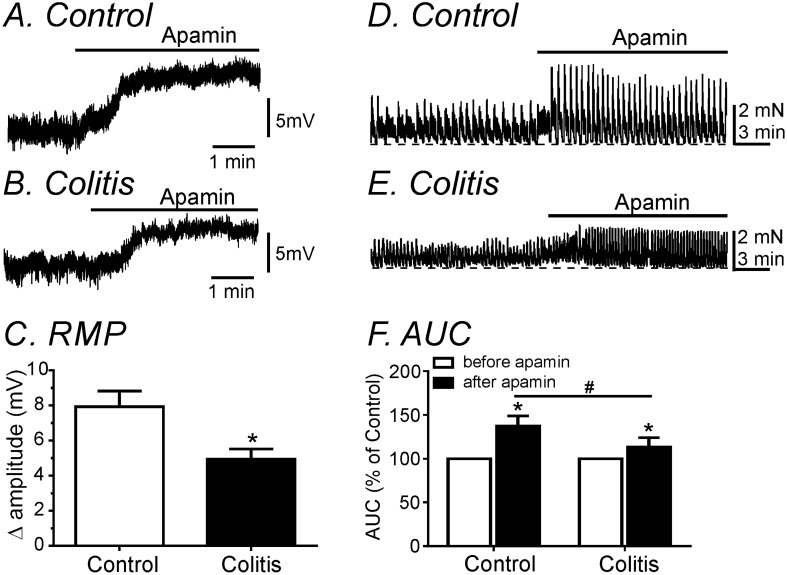
Comparisons of electrical and mechanical responses by apamin in control and DSS-treated colons. (**A,B**) Apamin induced less depolarization in DSS-treated colon compared to control colon. (**C**) The summarized data showed changes in amplitude of hyperpolarization in control and DSS-treated colons. (**D,E**) The contractile response by apamin was decreased in DSS-treated colon compared to control. (**F**) The summarized data of AUCs showed decreased the apamin sensitivity in DSS-treated colons. *Denotes *P* < 0.05 compared to before apamin in each group. ^#^Denotes *P* < 0.05 in comparison of apamin effects in control and DSS-treated colons.

SK channels (SK3) are expressed dominantly by PDGFRα^+^ cells in colonic muscles in comparison to SMCs^9,10^. Apamin, an SK channel antagonist, induced depolarization in untreated colonic muscles (Δ 7.9 ± 0.4 mV; n = 7; Fig. 4A,C). The effects of apamin were reduced in DSS-treated colons (Δ 4.9 ± 0.3 mV; *P* < 0.05; n = 7; Fig. 4B,C). In contractile experiments, responses to apamin (AUC) were reduced in DSS-treated colon in comparison to responses of untreated colonic muscles (138 ± 4.3% in control vs 113 ± 4.1% in DSS-treated colon, *P* < 0.05, n = 8 for each; Fig. 4D–F). These results suggest that sensitivity to apamin is decreased in colonic muscles from DSS-treated mice. We hypothesized that these effects could be due to decreased expression of SK3 channels in PDGFR α^+^ cells.

### Downregulation of *Pdgfra* and *Kcnn3* transcripts in colitis colon

**Figure 5:**
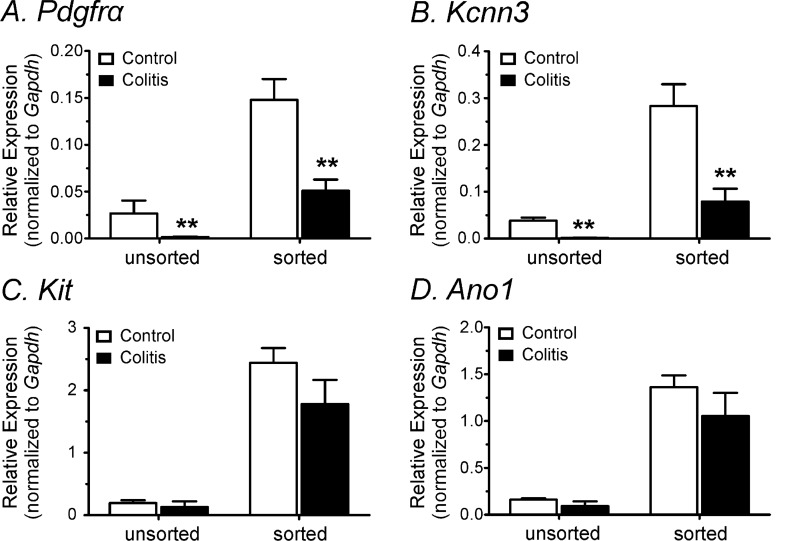
Comparison of transcriptional changes in unsorted and sorted cells from control and DSS-treated colons. (**A,B**) Quantitative analysis of *Pdgfra* and *Kcnn3* transcripts from unsorted cells and sorted PDGFRα^+^ cells showed downregulation of *Pdgfra* and *Kcnn3* in DSS-treated colon compared to control colon. (**C,D**) Both *Kit* and *Ano1* transcriptional expression from unsorted cells and sorted ICC were not significantly changed in control and DSS-treated colons. **Denotes *P* < 0.01 in panel (**A,B**).

Quantitative analysis of *Pdgfra* and *Kcnn3* in sorted PDGFRα^+^ cells was performed to determine whether transcriptional changes occur in DSS-treated colon (see “Methods” section). Colonic PDGFRα^+^ cells displayed downregulation of *Pdgfra* and *Kcnn3* after DSS-treatment (*P* < 0.01, n = 4 for both control and DSS-treated cells, Fig. 5A,B). Since *Kcnn3* is predominantly expressed in PDGFRα^+^ cells^9,10^, downregulation of *Pdgfra* and *Kcnn3* in unsorted cells from DSS-treated colon was likely due to downregulation in PDGFRα^+^ cells. Reduced expression of *Pdgfra* and *Kcnn3* is consistent with the differences in responses to apamin in control and colons from animals after DSS treatment (Fig. 4). We also examined whether expression of *Kit* and *Ano1* (markers of ICC) was changed in DSS-treated colon. Neither of these genes differed significantly in sorted ICC from control and DSS-treated colons (Fig. 5C,D).

### Change of excitatory response to thrombin

**Figure 6:**
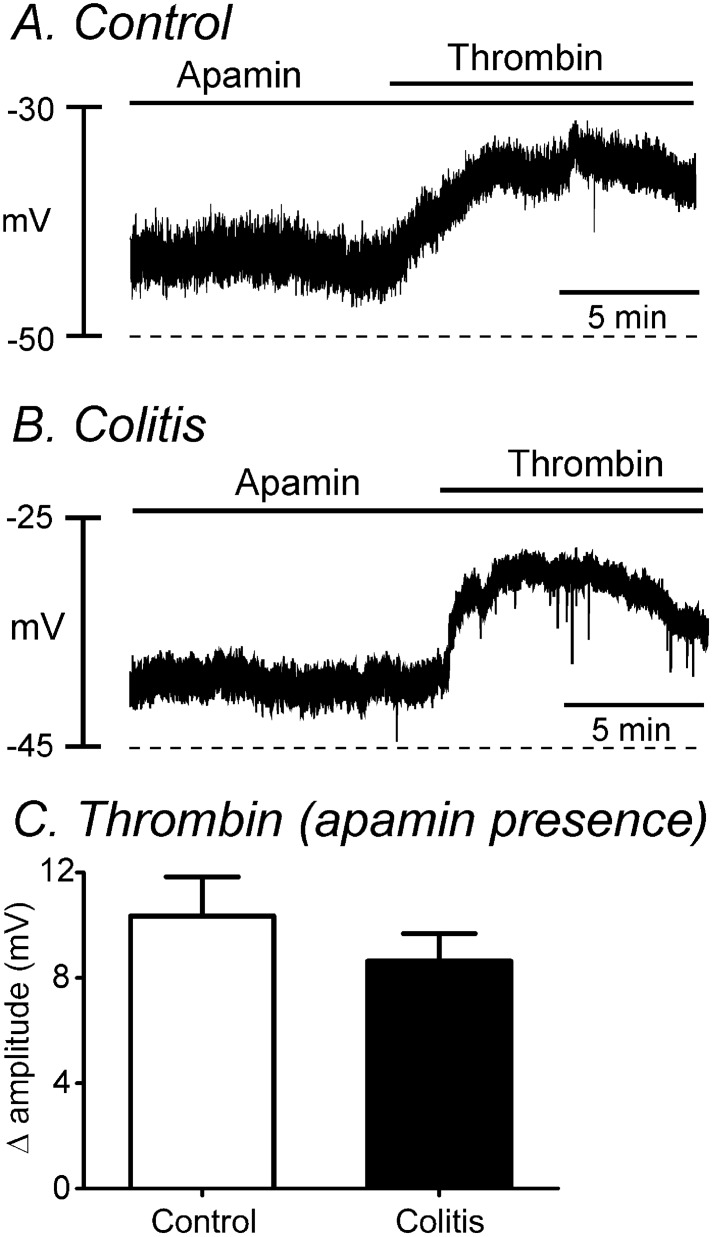
Comparison of electrical responses of thrombin in the presence of apamin in control and DSS-treated colons. (**A,B**) Thrombin application did not show a significant difference of depolarization between control (**A**) and DSS-treated (**B**) colons in the presence of apamin. (**C**) The summarized data did not show a significant change in membrane potentials by thrombin in the presence of apamin between two groups.

**Figure 7:**
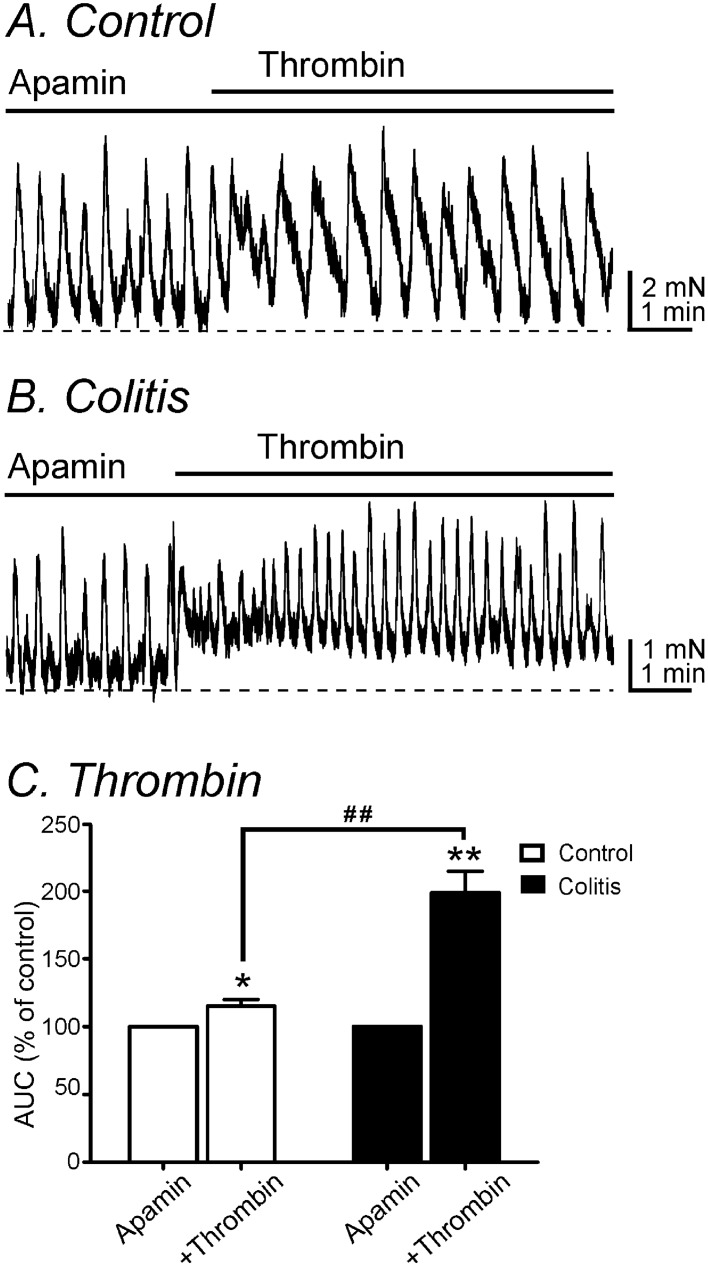
Comparison of mechanical responses of thrombin in the presence of apamin in control and DSS-treated colons. (**A,B**) Thrombin in DSS-treated colon (**B**) significantly increased the spontaneous contractions compared to the responses of thrombin in control colon (**A**) in the presence of apamin. (**C**) The summarized data showed changes in AUCs in response to thrombin. AUCs were normalized to the apamin response in each group. * and **Denote *P* < 0.05 and *P* < 0.01, respectively in each group. ^##^Denotes *P* < 0.01 in comparison with thrombin effect between control and DSS-treated colons.

We previously showed that the inhibitory effects of PAR1 are mediated through activation of SK3 channels in PDGFRα^+^ cells and excitatory phase of PAR responses is mediated mainly through activation of Ca^2+^-activated Cl^-^ channels (encoded by *Ano1*) in ICC^14^. Experiments were performed to investigate the excitatory phase of PAR responses in control and DSS-treated colons after blocking the initial inhibitory phase with apamin. After addition of apamin (300 nM), thrombin depolarized control muscles by 10.5 ± 1.6 mV (n = 6) and muscles from DSS-treated colons by 8.6 ± 1.0 mV (n = 5). The amplitude of depolarization by thrombin was not significantly different between control and DSS-treated colons (*P* = 0.39, Fig. 6A–C). The lack of differences in depolarization responses to the PAR1 agonist was consistent with the molecular data detecting no differences in *Kit* and *Ano1* expression in control and DSS-treated colons (see Fig. 5C,D). However, apamin, which blocked the initial relaxation caused by thrombin, increased spontaneous contractions, and the increase in contractions in response to thrombin was greatly augmented in DSS-treated colonic muscles. AUC of responses to thrombin in the presence of apamin increased from 115.1 ± 4.7% in control (n = 10) to 198.7 ± 15.9% in DSS-treated colons (n = 6, *P* < 0.01, Fig. 7). These experiments demonstrate differences in the electrical and mechanical responses to the PAR1 agonist after induction of DSS-colitis.

### Changes of elements involved in contraction

**Figure 8:**
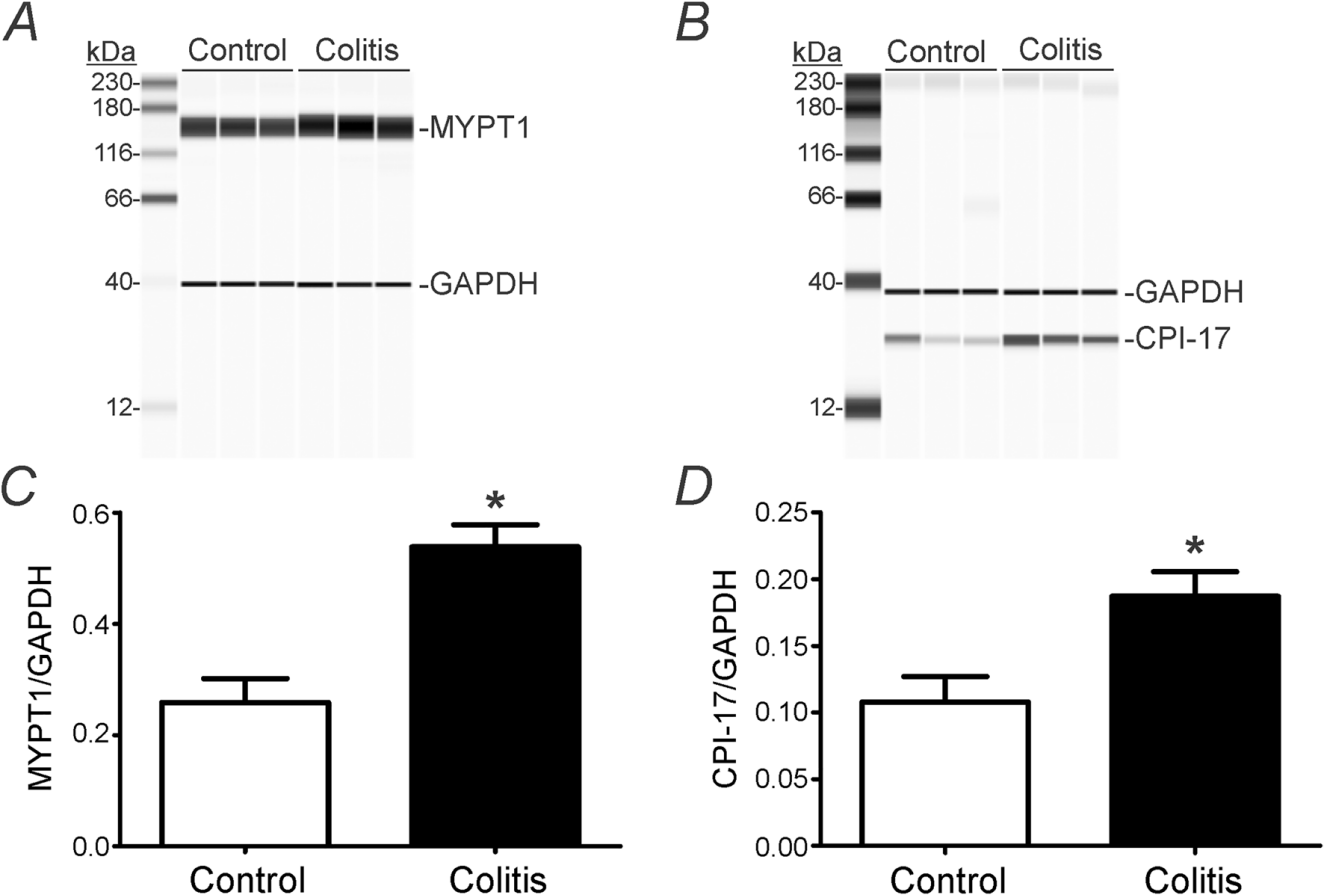
Changes in Ca^2+^ sensitization pathways in DSS-treated colon. (**A,B**) The protein expression of MYPT1 (**A**) and CPI-17 (**B**) was significantly increased in DSS-treated colon compared to those in control colon. (**C,D**) The summarized data showed significant changes in the ratios of MYPT1/GAPDH (**C**) and the ratios of CPI-17/GAPDH (**D**) between control colon and DSS-treated colon. *Denotes *P* < 0.05.

We hypothesized that the enhanced contractile responses observed after induction of DSS-colitis might be due to increased Ca^2+^-sensitization. Previously we showed that activation of PAR1 affects phosphorylation of MYPT1 (myosin phosphatase targeting subunit of MLCP) via G_12/13_/Rho Kinase pathway and CPI-17 (phosphatase inhibitor protein of 17 kDa) via G_q/11_/PLC/PKC pathway in colonic smooth muscle^15^. Expression of MYPT1 and CPI-17 proteins was increased significantly in DSS-treated colons compared to control colons. The ratios of MYPT1/GAPDH were 0.38 ± 0.03 in control colon (n = 4) and 0.59 ± 0.08 in DSS-treated colon (n = 4), respectively (*P* < 0.05, Fig. 8A,C). The ratios of CPI-17/GAPDH were in 0.11 ± 0.02 in control colon and 0.17 ± 0.01 in DSS-treated colon, respectively (*P* < 0.05, n = 4 for each; Fig. 8B,D). These results suggested that Ca^2+^-sensitization pathways are upregulated in DSS-treated colons, and consequently PAR1 activation enhanced contractile response.

### Colonic migrating motor complex in DSS-treated colons

**Figure 9:**
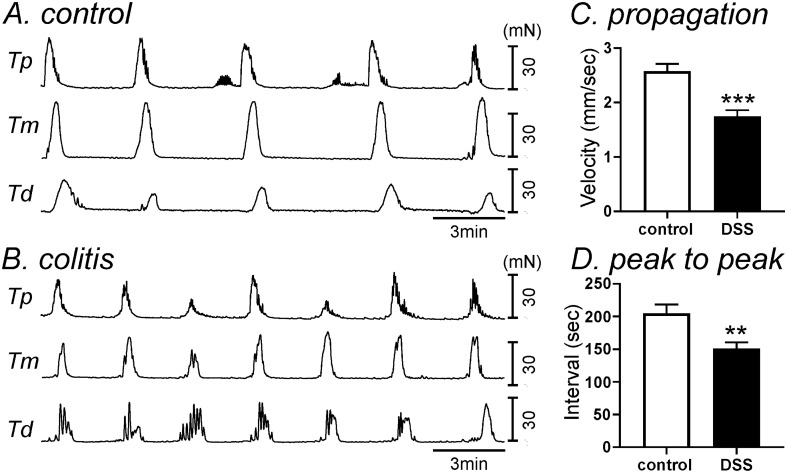
Colonic migrating motor complexes (CMMCs) in DSS-treated colon. (**A,B**) Tension was measured at proximal end of the colon (Tp), at the mid colon (Tm) and at the distal end (Td). The representative traces in control (**A**) and DSS-treated (**B**) colons showed that propagation velocity of CMMCs was significantly higher in DSS-treated colons compared to those in control colon (**C**), and peak to peak interval of CMMCs was also faster in DSS-treated colons compared to those in control colons (**D**). ** and ***Denote *P* < 0.01 and *P* < 0.001.

DSS-induced colitis can affect colonic migrating motor complexes (CMMCs). The propagation velocity of CMMCs in DSS-treated colon was 1.7 ± 0.11 mm/s (n = 10), which was significantly faster compared to control colons (2.6 ± 0.13 mm/s, n = 6, *P* < 0.001, Fig. 9A–C). To measure the frequency, we analyzed the peak to peak interval of CMMC. Peak to peak interval in DSS-treated colons was significantly shorter (151 ± 9.3 s, n = 10) in comparison to control interval (204 ± 13.4 s, n = 6, *P* < 0.01, Fig. 9A,B,D).

## Discussion

In the present study, we examined the effect of a PAR1 agonist on DSS-treated colons. DSS-treatment caused significant shortening of colons and decreased body weight. The transient hyperpolarization response to thrombin observed in control colonic muscles was greatly decreased in DSS-treated colons. Similarly, the initial relaxations observed in response to thrombin in control colonic muscles were changed by DSS-treatment to an increase in contractions without a relaxation phase. We reported previously that the initial hyperpolarization and relaxation responses to thrombin are due to activation of SK3 channels in PDGFRα^+^ cells^14^. In the present study we found that depolarization and contractile responses to apamin were decreased significantly after DSS-treatment in comparison to untreated colons. The depolarization phase of responses to thrombin is due to activation of ANO1 conductance in ICC^14^. Thus, we examined the effects of thrombin on membrane potentials in the presence of apamin. Thrombin induced less depolarizations in DSS-treated muscles than in control muscles in the presence of apamin. These differences were consistent with changes we observed in expression of key genes in PDGFRα^+^ cells and ICC. *Pdgfra* and *Kcnn3* were downregulated in cells from DSS-treated colons, but no statistically significant change was observed in *Kit* and *Ano1*. Seemingly at odds with the electrophysiological observations, contractile responses to thrombin increased significantly in colonic muscles after DSS-treatment and in the presence of apamin. This uncoupling between electrical and mechanical responses appeared to be due to enhanced expression of the main proteins responsible for Ca^2+^ sensitization responses in DSS colitis.

IBD results from multifactorial causes including genetic, immune, environmental and endogenous factors, such as defects in barrier function, vascular supply, or enteric nerve function^17^. Proteinases that activate PAR are found in increased concentrations in IBD patients^18,19^. Among a variety of chemically-induced animal models of colitis, DSS-induced colitis model has been used because it is simple and similar to human ulcerative colitis^20^. In the present study, we used DSS-induced colitis to examine how responses to a PAR1 agonist are remodeled in colitis.

PAR1 is a G protein-coupled receptor activated by thrombin. PAR1 activation mediates several effects of thrombin other than platelet aggregation, including inflammation. PAR1 activation induces changes in vascular tone, increased vascular permeability, and granulocyte chemotaxis^21,22^. In the gut, PAR1 is expressed by a variety of cell types, including enterocytes; endothelial cells; enteric neurons, SMCs, ICC and PDGFRα^+^ cells^14,15,23^. Intestinal motility is modulated by PAR1 activation. PAR1-activating peptides reduce spontaneous contractions or causes a biphasic response: initial relaxation followed by contraction in rat intestine^24,25^. In the mouse gastric fundus, responses to PAR1 activation are also biphasic characterized by relaxation that masks concomitant contractile effects^26^. PAR1 activation also causes biphasic contractile effects in the colon^14,15,23–25^. A study in vivo showed that administration of a PAR1 agonist increased gastrointestinal transit^27^, thus demonstrating that PAR1 activation can affect generalized motility patterns. The integrated effects of PAR1 activation on GI motility might depend on the cellular target that the PAR1 agonist first reaches. PDGFRα^+^ cells and ICC are both responsive to PAR agonists^14^. SMCs may also contribute to PAR responses via Ca^2+^-sensitization mechanisms intrinsic to these cells^15^. Electrophysiological pacemaker activity, post-junctional neural regulation and excitation–contraction coupling in SMCs that powers GI motility result from the combined actions of at least 3 types of cells, including SMCs, ICCs and PDGFRα^+^ cells. We have referred to these electrically coupled cells as the SIP syncytium^3^. For example, activation of SK3 channels, which are highly expressed in PDGFRα^+^ cells, causes hyperpolarization of cells in the SIP syncytium and decreases the excitability of SMCs^9,10^. In contrast, activation of ANO1 channels, which are expressed dominantly in ICC, depolarizes the cells of the SIP syncytium and increases the excitability in SMCs^28,29^. Thus, changes in expression of key conductances in SIP cells can influence excitability and possibly upset normal GI motility patterns. In this study, we dissected how DSS treatment affects cellular mechanisms that mediate responses to PAR1 and regulate contractile behaviors of colonic SMCs.

RMPs were depolarized in DSS-treated colonic muscles in comparison to colonic muscles from control mice. We also found that thrombin-induced hyperpolarization, which is inhibited by apamin and therefore mediated by activation of SK channels^14^, was also reduced in DSS-treated colons. Molecular data showed the downregulation of *Pdgfra* and *Kcnn3* which supported the functional data. Depolarization and the excitatory phase of PAR responses are mediated by ICC and due to activation of ANO1 channels, as this conductance is expressed in ICC and blocked by 5-nitro-2-(3-phenylpropyl-amino) benzoic acid (NPPB)^29,30^. Thrombin induces a transient relaxation in control colonic muscles. The relaxation phase of the thrombin response was absent in muscles from DSS-treated colon, and this was associated with downregulation of SK channels in PDGFRα^+^ cells. If the balance between depolarizing (activation of ANO1 channels in ICC) and hyperpolarizing (activation of SK3 channels in PDGFRα^+^ cells) is disrupted, as occurs in DSS colitis, one might expect greater depolarization and greater contractile responses to PAR1 activation. From the CMMC experiments we found that the propagation velocities and frequencies of CMMCs were increased in DSS-treated colon compare to control. This could be due to downregulation of SK channels in PDGFRα^+^ cells in DSS-treated colons. However, intestinal inflammation can affect intrinsic and extrinsic enteric neurons including degeneration of enteric nervous system, and alteration of glial cells^31^. In particular, PARs are expressed in the enteric nervous system which can also affect the membrane potentials in myenteric and submucosal neurons^32,33^. Therefore, it could require further investigation to interpret the results of CMMCs.

There have been several reports of colonic dysmotility and altered smooth muscle contractility in the mouse model of DSS colitis. Both increased and decreased contractility have been observed with DSS-induced colitis^34–37^. These contrasting smooth muscle contractile responses may result from differences in cytokine profiles. It has been postulated that Th2 cytokines primarily mediate colonic hypercontractility while Th1 cytokines mainly mediate colonic hypocontractility^36,38–40^. However, environmental factors including the hygienic condition of the vivarium also plays a critical role in the development of DSS-induced colitis^41^. Thus, it appears that the induced colonic contractile dysfunction depends on the inflammatory stimulus, the intestinal region, as well as the DSS source and lot number, molecular weight, concentration, duration, mouse strain and source, age, gender and body weight, and the vivarium environment. Greater contractile responses were observed in our experiments, but we did not observe statistically greater depolarization responses in muscles DSS-treated colons, even when residual SK3 channels were blocked by apamin. This suggests that other factors may be involved in shaping electrophysiological responses to thrombin. The increase in contractions in response to thrombin in DSS-treated colonic muscles also seemed to be due to additional factors, such as upregulation of Ca^2+^ sensitization mechanisms in SMCs.

PAR activation by thrombin also increases MYPT1 phosphorylation by ROCK, as previously reported^15^. Thrombin treatment significantly increased MYPT1 T853 phosphorylation in simian colonic muscles. CPI-17 T38 phosphorylation which is typically associated with Ca^2+^ influx. Apamin pretreatment inhibited hyperpolarization and increased CPI-17 T38 phosphorylation. Since CPI-17 can be phosphorylated by PKC^5,42,43^, Gö 6976, the inhibitor of Ca^2+^-dependent PKCs, blocked CPI-17 T38 phosphorylation in response to thrombin^15^. These previous reports suggest that thrombin also induces Ca^2+^ influx or release in SMCs and activates PKC to induce CPI-17 T38 phosphorylation. In the present study, we found that that expression of MYPT1 and CPI-17 proteins were increased in DSS-induced colitis, possibly increasing the Ca^2+^ sensitization mechanisms in SMCs. This remodeling may have contributed to the apparent uncoupling between membrane potential responses and contractions.

In conclusion, these studies showed that responses to the PAR1 agonist, thrombin, are remodeled in DSS colitis. Relaxation responses are abolished due to decreased expression of SK3 in PDGFRα^+^ cells and contractions are increased due to loss of the hyperpolarizing phase of the response to PAR1 activation and upregulation of proteins involved in Ca^2+^ sensitization. These cell specific alterations may provide a partial explanation for some of the dysmotility observed in colitis (Supplementary Information).

## Methods

### Animals

C57BL/6 mice for electromechanical experiments and Kit^copGFP/+^ and Pdgfra^tm11(EGFP)Sor^/J heterozygote mice (Jackson Laboratory, ME, USA) for molecular experiments were used as described previously^10,28^. All mice were housed at 20–25 °C temperature under a 12-h light/dark cycle with free access to water and food. Mice (6–10 weeks) were anesthetized by isoflurane (Baxter Healthcare, IL, USA) inhalation and sacrificed by dislocation. Animals were maintained in accordance with the NIH Guide for the Care and Use of Laboratory Animals. All methods are reported in accordance with ARRIVE guidelines. All procedures were approved by the Institutional Animal Use and Care Committee at the University of Nevada, Reno.

### Induction of DSS colitis in murine

Colitis was induced in C57BL/6 mice by giving 3% DSS salt, Reagent Grade (M.W. = 36–50 kDa; MP Biomedicals, CA, USA) in drinking water for 7 days. Control mice were given normal drinking water. Body weight and colon length were measured at 7th day. Clinical signs of inflammation in colon were recorded daily. The disease activity index (DAI) of colitis mice was scored daily for each mouse as described previously^44^, based on body weight loss, occult blood, and stool consistency.

### Isometric force recording

Recording of colonic motility were performed as described previously^14^. Briefly, muscle strips (1 cm length × 0.2 cm width) of proximal colon were attached to an isometric force transducer (Fort 10, WPI, FL, USA) in a 5 ml organ bath containing oxygenated (97% O_2_ and 3% CO_2_) Krebs Ringer buffer (KRB) at 37 ± 0.5 °C. Muscle strips were stabilized for 60 min. Force of 1–2 mN was applied at rest to set the muscles at optimum lengths followed by equilibrating for 60 min. Mechanical responses were recorded with a computer running PowerLab 4/35 (AD Instruments, CO, USA). AUC during 2 min recordings was measured, and this parameter was compared before and after drugs. Muscle strips were pre-treated with tetrodotoxin (TTX, 1 µM) for 10 min before adding PAR agonist to decontaminate the responses due to neural effects.

### Transmembrane potential recording

The membrane potential of colonic SMCs was measured using intracellular recordings as described previously^14^. Briefly, muscle strips (0.3 cm width × 0.6 cm length) of proximal colon were prepared and SMCs were impaled with microelectrodes having resistances of 80–100 MΩ perfusing with oxygenated and pre-warmed (37 ± 0.5 °C) KRB. Transmembrane potential was measured with a high input impedance amplifier (WPI Duo 773, FL, USA) and recorded with a computer running AxoScope (Molecular Device, Foster city, CA). Experiments were performed in the presence of TTX (1 µM) and nicardipine (1 µM) to exclude neural influence and decrease spontaneous contraction to keep impalement.

### Measurements of colonic migrating motor complexes (CMMCs)

Whole colon was extracted from body to measure CMMCs. In order to secure whole colon to the floor of the organ, a glass microelectrode pipette (Sutter Instrument Co., Novato, CA, USA) was inserted through the lumen with using U‐shaped pins at each end as previously described^45^. Three isometric tension transducers (model FT03, Grass, MA, USA) were attached by suture silk at regular intervals to the colonic wall in order to measure the tension of the circular muscle; the silk was glued to the colon by a bead of Vetbond (*n*‐butyl cyanoacrylate; St Paul, MN, USA) and the initial resting tension was set to 10 mN. Three tension transducers were placed at 10–15 mm from the proximal end of the colon (Tp), at the mid colon (Tm) and at 10–15 mm from the distal end (Td). Tissues were equilibrated for 1–2 h until regular spontaneous CMMCs were observed. Labchart (version 6, AD instruments, USA) were used for acquisition. Clampfit (v10.1, Molecular Device, USA) was used for analysis.

### Molecular studies

Total RNA was isolated from ICC of Kit^copGFP/+^ mice, PDGFRα^+^ cells of Pdgfra^tm11(EGFP)Sor^/J heterozygote mice which were purified by fluorescence-activated cell sorting (Becton Dickinson FACSAria using the blue laser (488 nm) and the GFP emission detector; 530/30 nm) and unsorted cells which were total cell population before cell sorting. Total RNA isolation, cDNA preparation and amplification of murine colonic muscle apparatus were performed as previously reported^9^. The relative expression levels of *Pdgfra, Kcnn3, Kit and Ano1* were determined by real-time quantitative PCR performed on an ABI PRISM™ 7000 sequence detector using SYBR® Green chemistry (Applied Biosystems, CA). Standard curves were generated for each gene and the constitutively expressed Gapdh from regression analysis of the mean values of RT-PCRs for the log_10_ diluted cDNA. Each cDNA sample was tested in triplicate and cDNA was obtained from 4 different murine colons. The reproducibility of the assay was tested by analysis of variance comparing repeat runs of samples, and the mean values generated at individual time points were compared by Student’s *t* test.

### Automated capillary electrophoresis and immunoblotting with Wes Simple Western

Colonic muscles were treated for electrophoresis as described previously^46^, and then tissues were homogenized in 0.5 ml lysis buffer and centrifuged at 16,000×*g* at 4 °C for 10 min. Protein expression levels were measured and analyzed according to the Wes User Guide using a Wes Simple Western instrument from ProteinSimple (San Jose, CA, USA). The procedure followed as described previously^47^. The protein samples were mixed with the Fluorescent 5× Master Mix (ProteinSimple) and then heated at 95 °C for 5 min. Boiled samples and primary antibodies (mouse anti-CPI-17 (PPP1R14A) (sc-48406; 1:200) and rabbit anti-MYPT1 (PPP1R12A) (sc-25618; 1:200)) were loaded into the Wes plate (Wes 12–230 kDa Pre-filled Plates with Split Buffer, ProteinSimple) with a Biotinylated protein ladder, horseradish peroxidase-conjugated anti-rabbit or anti-mouse secondary antibodies, Luminol-peroxide and washing buffer (ProteinSimple). The plates and capillary cartridges were loaded into the Wes instrument, and protein separation, antibody incubation and imaging were performed using default parameters (see also supplementary file). Compass software (ProteinSimple) was used to acquire the data, and to generate image reconstruction and chemiluminescence signal intensities. The protein levels are expressed as the area of the peak chemiluminescence intensity.

### Solutions and drugs

In mechanical and electrical recordings, experiments were performed in oxygenated KRB (in mmol/l): 120 NaCl; 5.9 KCl; 1.2 MgCl_2_; 15.5 NaHCO_3_; 1.2 NaH_2_PO_4_; 11.5 dextrose; and 2.5 CaCl_2_ with 7.3–7.4 pH at 37.0 ± 0.5 °C. Thrombin was purchased from Alfa Aesar (Haverhill, MA, USA) and TTX, atropine and N^G^-Nitro-l-arginine methyl ester hydrochloride (L-NAME) were purchased from Sigma (St Louis, MO, USA) and apamin was from Santa Cruz biotechnology, Inc (Dallas, TX, USA).

### Statistical analysis

Data are described as the means ± SEM. The analysis of data differences between groups was performed using Student’s *t*-test or one-way ANOVA followed by post hoc test when needed. *P*-values less than 0.05 were taken as statistically significant and n-values corresponded to the number of animals that were used in the indicated experiments.

## Supplementary Information

Supplementary Information.

## Data Availability

The data can be available from the correspondence authors upon reasonable request.

## References

[1] Bharucha AE (2008). Lower gastrointestinal functions. Neurogastroenterol. Motil..

[2] Rogler G (2014). Chronic ulcerative colitis and colorectal cancer. Cancer Lett..

[3] Sanders KM, Koh SD, Ro S, Ward SM (2012). Regulation of gastrointestinal motility-insights from smooth muscle biology. Nat. Rev. Gastroenterol. Hepatol..

[4] Sanders KM, Kito Y, Hwang SJ, Ward SM (2016). Regulation of gastrointestinal smooth muscle function by interstitial cells. Physiology (Bethesda).

[5] Sanders KM, Ward SM, Koh SD (2014). Interstitial cells: Regulators of smooth muscle function. Physiol. Rev..

[6] Bernardini N (2012). Immunohistochemical analysis of myenteric ganglia and interstitial cells of Cajal in ulcerative colitis. J. Cell Mol. Med..

[7] Blair PJ, Rhee PL, Sanders KM, Ward SM (2014). The significance of interstitial cells in neurogastroenterology. J. Neurogastroenterol. Motil..

[8] Nakahara M (2002). Dose-dependent and time-limited proliferation of cultured murine interstitial cells of Cajal in response to stem cell factor. Life Sci..

[9] Kurahashi M (2011). A functional role for the 'fibroblast-like cells' in gastrointestinal smooth muscles. J. Physiol..

[10] Kurahashi M, Mutafova-Yambolieva V, Koh SD, Sanders KM (2014). Platelet-derived growth factor receptor-alpha-positive cells and not smooth muscle cells mediate purinergic hyperpolarization in murine colonic muscles. Am. J. Physiol. Cell Physiol..

[11] Gallego D, Hernandez P, Clave P, Jimenez M (2006). P2Y1 receptors mediate inhibitory purinergic neuromuscular transmission in the human colon. Am. J. Physiol. Gastrointest. Liver Physiol..

[12] Mutafova-Yambolieva VN (2007). Beta-nicotinamide adenine dinucleotide is an inhibitory neurotransmitter in visceral smooth muscle. Proc. Natl. Acad. Sci. U.S.A..

[13] Song NN (2018). Diabetes-induced colonic slow transit mediated by the up-regulation of PDGFRalpha(+) cells/SK3 in streptozotocin-induced diabetic mice. Neurogastroenterol. Motil..

[14] Sung TS (2015). Protease-activated receptors modulate excitability of murine colonic smooth muscles by differential effects on interstitial cells. J. Physiol..

[15] Sung TS (2018). The functional role of protease-activated receptors on contractile responses by activation of Ca(2+) sensitization pathways in simian colonic muscles. Am. J. Physiol. Gastrointest. Liver Physiol..

[16] Sato K, Ninomiya H, Ohkura S, Ozaki H, Nasu T (2006). Impairment of PAR-2-mediated relaxation system in colonic smooth muscle after intestinal inflammation. Br. J. Pharmacol..

[17] Kirsner JB (2000). Inflammatory Bowel Disease.

[18] Bustos D (1998). Colonic proteinases: Increased activity in patients with ulcerative colitis. Medicina (B Aires).

[19] Kjeldsen J, Lassen JF, Brandslund I, de Muckadell OBS (1998). Markers of coagulation and fibrinolysis as measures of disease activity in inflammatory bowel disease. Scand. J. Gastroenterol..

[20] Chassaing B, Aitken JD, Malleshappa M, Vijay-Kumar M (2014). Dextran sulfate sodium (DSS)-induced colitis in mice. Curr. Protoc. Immunol..

[21] Coughlin SR, Camerer E (2003). PARticipation in inflammation. J. Clin. Investig..

[22] Vergnolle N, Wallace JL, Bunnett NW, Hollenberg MD (2001). Protease-activated receptors in inflammation, neuronal signaling and pain. Trends Pharmacol. Sci..

[23] Corvera CU (1999). Thrombin and mast cell tryptase regulate guinea-pig myenteric neurons through proteinase-activated receptors-1 and -2. J. Physiol..

[24] Mule F, Baffi MC, Cerra MC (2002). Dual effect mediated by protease-activated receptors on the mechanical activity of rat colon. Br. J. Pharmacol..

[25] Mule F, Baffi MC, Falzone M, Cerra MC (2002). Signal transduction pathways involved in the mechanical responses to protease-activated receptors in rat colon. J. Pharmacol. Exp. Ther..

[26] Cocks TM, Sozzi V, Moffatt JD, Selemidis S (1999). Protease-activated receptors mediate apamin-sensitive relaxation of mouse and guinea pig gastrointestinal smooth muscle. Gastroenterology.

[27] Kawabata A (2001). In vivo evidence that protease-activated receptors 1 and 2 modulate gastrointestinal transit in the mouse. Br. J. Pharmacol..

[28] Zhu MH (2009). A Ca(2+)-activated Cl(-) conductance in interstitial cells of Cajal linked to slow wave currents and pacemaker activity. J. Physiol..

[29] Zhu MH (2011). Muscarinic activation of Ca2+-activated Cl-current in interstitial cells of Cajal. J. Physiol..

[30] Hwang SJ (2009). Expression of anoctamin 1/TMEM16A by interstitial cells of Cajal is fundamental for slow wave activity in gastrointestinal muscles. J. Physiol..

[31] Bernardazzi C, Pego B, de Souza HS (2016). Neuroimmunomodulation in the gut: Focus on inflammatory bowel disease. Mediat. Inflamm..

[32] Linden DR, Manning BP, Bunnett NW, Mawe GM (2001). Agonists of proteinase-activated receptor 2 excite guinea pig ileal myenteric neurons. Eur. J. Pharmacol..

[33] Reed DE (2003). Mast cell tryptase and proteinase-activated receptor 2 induce hyperexcitability of guinea-pig submucosal neurons. J. Physiol..

[34] Eichele DD, Kharbanda KK (2017). Dextran sodium sulfate colitis murine model: An indispensable tool for advancing our understanding of inflammatory bowel diseases pathogenesis. World J. Gastroenterol..

[35] Ihara E, Chappellaz M, Turner SR, MacDonald JA (2012). The contribution of protein kinase C and CPI-17 signaling pathways to hypercontractility in murine experimental colitis. Neurogastroenterol. Motil..

[36] Ohama T (2008). Downregulation of CPI-17 contributes to dysfunctional motility in chronic intestinal inflammation model mice and ulcerative colitis patients. J. Gastroenterol..

[37] Sato K (2007). Involvement of CPI-17 downregulation in the dysmotility of the colon from dextran sodium sulphate-induced experimental colitis in a mouse model. Neurogastroenterol. Motil..

[38] Akiho H, Deng Y, Blennerhassett P, Kanbayashi H, Collins SM (2005). Mechanisms underlying the maintenance of muscle hypercontractility in a model of postinfective gut dysfunction. Gastroenterology.

[39] Kiesler P, Fuss IJ, Strober W (2015). Experimental models of inflammatory bowel diseases. Cell Mol. Gastroenterol. Hepatol..

[40] Ohama T, Hori M, Ozaki H (2007). Mechanism of abnormal intestinal motility in inflammatory bowel disease: How smooth muscle contraction is reduced?. J. Smooth Muscle Res..

[41] Chassaing B, Aitken JD, Malleshappa M, Vijay-Kumar M (2014). Dextran sulfate sodium (DSS)-induced colitis in mice. Curr. Protoc. Immunol..

[42] Perrino BA (2016). Calcium sensitization mechanisms in gastrointestinal smooth muscles. J. Neurogastroenterol. Motil..

[43] Eto M, Senba S, Morita F, Yazawa M (1997). Molecular cloning of a novel phosphorylation-dependent inhibitory protein of protein phosphatase-1 (CPI17) in smooth muscle: Its specific localization in smooth muscle. FEBS Lett..

[44] Cooper HS, Murthy SN, Shah RS, Sedergran DJ (1993). Clinicopathologic study of dextran sulfate sodium experimental murine colitis. Lab. Investig..

[45] Koh SD (2022). Propulsive colonic contractions are mediated by inhibition-driven poststimulus responses that originate in interstitial cells of Cajal. Proc. Natl. Acad. Sci. U.S.A..

[46] Bhetwal BP (2013). Ca2+ sensitization pathways accessed by cholinergic neurotransmission in the murine gastric fundus. J. Physiol..

[47] Xie Y (2018). A role for focal adhesion kinase in facilitating the contractile responses of murine gastric fundus smooth muscles. J. Physiol..

