# Supplementary material for: Altered functional responses by PAR1 agonist in murine dextran sodium sulphate-treated colon
Source: Sci Rep. 2022 Oct 6;12:16746. doi: 10.1038/s41598-022-21285-2 (PMC9537183; doi:10.1038/s41598-022-21285-2)
Supplement: Supplementary file 1 — Supplementary Information. [file 41598_2022_21285_MOESM1_ESM.docx]

**SUFPPLEMENTAL INFORMATION**

The Wes ProteinSimple system is a capillary western blot method that has the advantage of being a more sensitive and automated approach for protein detection while providing comparable results to traditional western blotting (Chen et al, 2013; Lu et al, 2018).

Briefly, similar to SDS-PAGE, the Wes separates lysates (boiled in SDS-sample buffer) through individual microcapillary tubes; the separated proteins are fixed in place within each tube, the separation matrix is washed out, and then the primary antibodies are added to each tube, followed by the HRP-conjugated secondary antibodies, and then finally luminol peroxide to develop the signals. The Wes Compass software then scans each tube for chemiluminescence peak detection, and generates a gel image (bitmap) of the results. Similar to other image acquisition and processing software, the gel image bitmap can be evaluated with different exposure times; however, different exposure times cannot be used for different microcapillary cartridges; that is, all the lanes in the gel image represent the same exposure time for each individual microcapillary cartridge. When deemed necessary, image processing (such as changing brightness and contrast) is carried out using Photoshop, and is applied equally across the entire image and is applied equally to controls.  Each bitmap is then copied and pasted into CorelDraw to add labels. Below are provided the original uncropped bitmap image, from which Fig 8A and 8B were generated. This bitmap image was not processed with Photoshop.

Image for Fig 8A and 8B

Chen, J. Q., Heldman, M. R., Herrmann, M. A., Kedei, N., Woo, W., Blumberg, P. M. & Goldsmith, P. K. (2013) Absolute quantitation of endogenous proteins with precision and accuracy using a capillary Western system. *Anal Biochem*, 442(1), 97-103.

Lu, J., Allred, C. C. & Jensen, M. D. (2018) Human adipose tissue protein analyses using capillary western blot technology. *Nutr Diabetes*, 8(1), 26.
